# A Comparison of the Different Animal Models of Fetal Alcohol Spectrum Disorders and Their Use in Studying Complex Behaviors

**DOI:** 10.3389/fped.2014.00093

**Published:** 2014-09-03

**Authors:** Anna R. Patten, Christine J. Fontaine, Brian R. Christie

**Affiliations:** ^1^Division of Medical Sciences, University of Victoria, Victoria, BC, Canada; ^2^Department of Biology, University of Victoria, Victoria, BC, Canada; ^3^Program in Neuroscience, The Brain Research Centre, University of British Columbia, Vancouver, BC, Canada; ^4^Department of Cellular and Physiological Sciences, University of British Columbia, Vancouver, BC, Canada

**Keywords:** FASD, behavior, animal models, alcohol, prenatal ethanol exposure

## Abstract

Prenatal ethanol exposure (PNEE) has been linked to widespread impairments in brain structure and function. There are a number of animal models that are used to study the structural and functional deficits caused by PNEE, including, but not limited to invertebrates, fish, rodents, and non-human primates. Animal models enable a researcher to control important variables such as the route of ethanol administration, as well as the timing, frequency and amount of ethanol exposure. Each animal model and system of exposure has its place, depending on the research question being undertaken. In this review, we will examine the different routes of ethanol administration and the various animal models of fetal alcohol spectrum disorders (FASD) that are commonly used in research, emphasizing their strengths and limitations. We will also present an up-to-date summary on the effects of prenatal/neonatal ethanol exposure on behavior across the lifespan, focusing on learning and memory, olfaction, social, executive, and motor functions. Special emphasis will be placed where the various animal models best represent deficits observed in the human condition and offer a viable test bed to examine potential therapeutics for human beings with FASD.

## Introduction

Ethanol is a teratogen that disrupts normal development. The use of animal models to study how ethanol affects the development of offspring in animal models can be traced back to the late 1970s, when several groups began to study ethanol’s effects on the development of laboratory rats. How this agent affects the development of the brain and body remains a subject of intense investigation, and it is worthwhile to appreciate some of the guiding principles that drive this field of investigation, particularly as they relate to the choice of animal model to be used. The choice of animal to be used can be critical, as genetic susceptibility can play a major role in determining ethanol’s effects. For instance, in some species any teratogenic effects may be induced with relatively low doses, while other species may be more impervious to the effects of ethanol. Second, one has to appreciate that the developmental stage of the organism at the time of exposure can play a significant role in how ethanol disrupts development. There are critical periods of *in utero* growth and development where certain brain or organ systems will be undergoing rapid development and thus be more prone to damage by teratogenic agents. Third, understanding how teratogenic agents act on proteins and signaling systems in developing cells will be key to understand how ethanol can initiate sequences of abnormal development at a cellular level. Certain animal model systems will lend themselves more readily to these sorts of experiments, depending on the nature and complexity of the question being asked. Fourth, it is critical to understand the nature of the agent itself, as the route and degree of maternal exposure, as well as the rate of placental transfer and systemic absorption are key factors in determining how severely ethanol will affect organism. A fifth consideration is that one needs to be vigilant for the four major signs of deviant development (death, malformation, growth retardation, and functional defect) when examining the effects of ethanol in any animal model. Functional defects may occur without any significant malformation or growth retardation. Finally, it should be clear that any disruptions in normal development will likely increase in frequency and degree as dosage increases. Ethanol is unusual in that it is both lipid and water soluble, so when it is consumed by pregnant females it can rapidly transit the placental membrane and directly affect the fetus (1). With these considerations in mind, let us briefly examine what we know of how ethanol affects the human condition.

Fetal alcohol syndrome (FAS) is the most severe disorder that results from prenatal ethanol exposure (PNEE). FAS is a disorder characterized by facial dysmorphologies (such as midfacial hypoplasia, wide spaced eyes, and a smooth philtrum), growth retardation, and CNS dysfunction resulting in cognitive, motor, and behavioral problems (2). Since FAS was first defined in the 1970s (3, 4) researchers have become more aware that the damage caused by ethanol can vary due to the timing, frequency, and volume of ethanol consumed. In addition, genetics and the metabolism of the mother can also play a role (5), leading to significant variability in the severity and symptoms associated with PNEE. Understanding that variability in genetic make-up, and variability in the timing and dose of ethanol consumption, can impact how ethanol affects development has resulted in the umbrella term FASD being adopted to refer to any condition that results from PNEE. This term encompasses children who exhibit varying degrees of central nervous system (CNS) dysfunction including alcohol-related birth defects (ARBD) and alcohol-related neurological disorders (ARND) that result from PNEE. These conditions often lack the facial dysmorphology needed to meet the diagnostic criteria for FAS, but are never-the-less the result of exposure to this teratogen during development (2, 6).

Although we have been aware that ethanol is a teratogen since the 1970s, there are still large numbers of children affected by PNEE (7). In part, this is because many women do not realize they are pregnant in the first trimester and continue binge drinking (8, 9). Furthermore, in many countries a significant percentage of pregnant women continue to consume ethanol throughout pregnancy – 10–20% in the USA, 40% in Uruguay, and 50% in some parts of Italy (10–12). In the United States, the lifetime cost for an individual suffering from FAS may be as high as $2 million. The majority of these costs are required for special education, medical, and mental health treatment (13). Currently in Canada, the annual cost of health care problems associated with PNEE is over $5 billion (14).

### Cognitive symptoms

Prenatal ethanol exposure can lead to a host of cognitive impairments. The severity and nature of these impairments depends on the amount and duration of alcohol consumption during pregnancy (4, 15–19). Children with FASD display a multitude of neuropsychological issues including deficits in mathematical ability, verbal fluency, memory, attention, learning capabilities, executive function, fine motor control, and social interaction, with the number of issues and the extent of damage varying from child to child (15, 17, 19, 20). To be diagnosed with an intellectual disability, generally a child must have an intelligence quotient (IQ) two or more standard deviations below the norm, roughly equating a score below 70, while scores between 71 and 85 are considered to represent borderline intellectual function [DSM V (21)]. Children with FAS generally have IQs estimated in the low 70s but the range can be anywhere between 20 and 120 (16, 22). Children without the complete FAS diagnosis (but with the FASD diagnosis) also generally have low IQs with averages in the low 80s (23).

### Underlying mechanisms of PNEE damage

Because of the variety of deficits that occur with FASD it can be hard to pinpoint the structural and functional changes that occur in the developing CNS and to identify how they relate to a particular behavioral disorder. Multiple brain regions are affected, and the areas and extent of damage depend on the amount and timing of ethanol ingestion. A number of molecular mechanisms may play a role, and these may be activated at different stages of development or at different dose thresholds of exposure [see Ref. (24, 25) for review]. These include disrupted cell energetics (26–30); cell cycle interference, and a deregulation of developmental timing (31–35); alterations in retinoic acid signaling (36); interference with cell and growth factor signaling (37–39); and apoptosis (38, 40, 41). Furthermore, many neurotransmitters, adhesive molecules, transcription factors, and trophic factors can be either up- or down-regulated by PNEE, making FASD a very complex syndrome [see Ref. (24) for review].

### Objectives

The study of human subjects is invaluable for FASD research, however, epidemiological studies are often limited by ethical constraints and a multitude of confounding variables including multi-substance abuse, diet, maternal health, and genetic or socioeconomic background (25, 42). It is also difficult to get reliable estimates on the amount and timing of ethanol exposure when self-reporting from the mothers is necessary. Due to these constraints, studies in human beings have focused on finding biomarkers of PNEE in fetal meconium (43) and hair samples [see Ref. (44) for review] through the presence of fatty acid esters [see Ref. (45) for review].

Animal models provide a simple and reliable method to study the effects of alcohol on the developing brain and eliminate many of the obvious confounds associated with human studies. These models can be used to understand the mechanism of the toxic effects of ethanol on the developing brain and to develop and test potential therapies to combat these effects. Animal models enable the experimenter to manipulate social and behavioral contexts; to control for stress and nutritional variables; and to do all of this in an organism that has a condensed lifespan in relation to human beings. In this review, the different animal models of FASD will be outlined and the advantages and disadvantages of each model will be discussed. This will be followed by an in depth discussion of the cognitive deficits that have been observed in the animal models of PNEE.

## Factors to Consider When Modeling FASD

Because FASD is such a complex disorder and there are so many facets to explore, there are many factors to consider when choosing an appropriate model for a particular study. The level of intoxication achieved during brain development, the particular period of brain development that is to be targeted (first, second, or third trimester), the pattern of administration (chronic or acute) and the route of administration (ingestion, injection or inhalation) can all be manipulated.

There are also a wide variety of animal models available for FASD research ranging from the simple (*Caenorhabditis elegans, Drosophila*, zebrafish, *Xenopus*) to the complex (rodents and non-human primates). Rodents are by far the most common model employed, with rat, mouse, and guinea pig models utilized in laboratories throughout Canada and the USA. All these models have been shown to mimic at least some aspects of the human condition including the craniofacial abnormalities (46, 47), growth retardation (48–50), physiological impairments (51–53), and cognitive deficits (42, 54–56) reviewed in Ref. (42, 57). However, similar to the variability that is observed in human beings, there is no single animal model that mimics all the features of FAS and/or FASD. When deciding on which model to utilize, it is pertinent to choose based on the research question to be examined. In this section, we will first discuss the pertinent factors to consider when designing a study of PNEE followed by a breakdown of each of the animal models, with the major strengths and limitations of each method considered. It is important to note that we have limited our discussion of animal models to simple systems (*C. elegans, Xenopus*, and zebrafish) and more sophisticated rodent and non-human primate models. There is also FASD research being conducted using chicken (58) and sheep (59–62) models, however, because there is little behavioral analysis using these models we have omitted them from our review.

### Blood alcohol concentration

In Canada and USA, a blood alcohol concentration (BAC) of 80 mg/dl is considered legally intoxicated. If a 150 lb pregnant female consumes six alcoholic beverages, or a bottle of wine in a 2 h period a BAC of 200 mg/dl would be reached. In human studies, the BAC data from the mothers are generally not available, however, estimates suggest that BACs of over 200 mg/dl may be responsible for the severe FAS phenotype (63), while lower BACs may produce milder forms of FASD. Despite the lack of BAC data in human beings this measure is often used to compare exposure levels across species. This is because the absolute dose of ethanol administered (in gram of ethanol/kilogram) can vary greatly from species to species (42) so the BAC is a more reliable measure of intoxication.

Most animal studies use a dosage of alcohol exposure that produces a BAC in the range of 100–400 mg/dl (i.e., moderate to binge-like levels of exposure). The peak BAC achieved will depend on both the dose and pattern of exposure (64, 65). In order to achieve a low to moderate BAC (80–150 mg/dl), experimenters normally employ either liquid diets, voluntary drinking paradigms, or vapor inhalation (see Route of Administration). Higher binge-like BACs (>200 mg/dl) are normally achieved using either oral intubation (gavage) or direct injections (see Route of Administration). Higher BACs are generally associated with increased neurotoxicity, and even the administration of a single high dose of ethanol during the period of brain development can cause significant structural impairments if the BAC achieved is sufficiently high (66, 67). Low to moderate BACs can also cause significant neuronal damage, and while longer exposure periods (i.e., throughout gestation) are usually used with these models (68–70), shorter exposure can still cause significant deficits (31). Thus, continuous low-level exposure to ethanol may be as damaging as a single high-level exposure, though the types of deficits incurred may differ. The deficits observed with either mode of administration can be affected by the timing of ethanol exposure.

### Developmental timing of ethanol exposure

The timing of ethanol exposure can greatly influence the outcome of the fetus. The mammalian brain develops in six major phases, commencing with neural cell genesis, followed by neuronal migration, glial cell proliferation, axon and dendrite proliferation, synaptogenesis, extensive pruning and cell death, and finally myelination of the axons (71). These steps occur in all regions of the brain but different regions develop at different times depending on their caudal or rostral location. Brain development is a dynamic process and it is therefore important to consider the developmental timing of alcohol exposure when choosing a model, based on regional and temporal windows of vulnerability. Gestation and development in simple vertebrates (e.g., *Xenopus, C. elegans*, or zebrafish) and even rodents (mice, rats, guinea pigs) is significantly different from human beings. The human gestation period is characterized by three trimesters, all of which occur prenatally. In the first trimester, formation of the neural tube and gastrulation occurs and in the second trimester cell proliferation and migration occur profusely. Finally, in the third trimester a “brain growth spurt” occurs, which is characterized by large amounts of growth and differentiation (72).

Rodents are the most commonly used animal model used for FASD research (see Rodents), however, their gestational period is much shorter than that of human beings (18–23 days for mice/rats; 68 days for guinea pigs), and a significant amount of brain development occurs following birth in these species (73, 74). The development period of the rodent brain is also divided into trimester equivalents; in the guinea pig, the three trimester equivalents largely occur prenatally, and therefore more closely resemble brain development in human beings. In rats and mice, the first trimester equivalent is from gestational day (GD) 1–10, the second trimester equivalent corresponds to GD 10–20 (just prior to birth) and the third trimester equivalent and “brain growth spurt” occurs following birth [from postnatal day (PND) 1 to 10] (75). In order to expose the brain to alcohol through all three trimester equivalents, alcohol must be administered to neonate pups (via oral intubation; see Ingestion), and the mechanisms of exposure, absorption, and elimination of this substance are significantly different during the prenatal and postnatal periods. For example, ethanol metabolizing enzymes, such as alcohol dehydrogenase, are only at 25% of adult levels at birth (76). Normally, the fetus is partially protected by the mothers’ capacity to metabolize ethanol, so in rodent pups it is routinely reported that higher BAC levels are produced in neonates with lower alcohol doses than those used in pregnant dams (77–80).

### Route of administration

There are several different methods that can be used to administer ethanol during pregnancy. In invertebrates and simple vertebrates (*C. elegans, Xenopus*, zebrafish), alcohol exposure is usually by bath application (see Simple Systems). In more complex models such as those using rodents and primates, there are three major methods of ethanol administration employed: ingestion (through diet, water, or intubation), injection, or inhalation [for additional reviews see Ref. (25, 81, 82)].

#### Ingestion

##### Dietary

**Figure 1:**
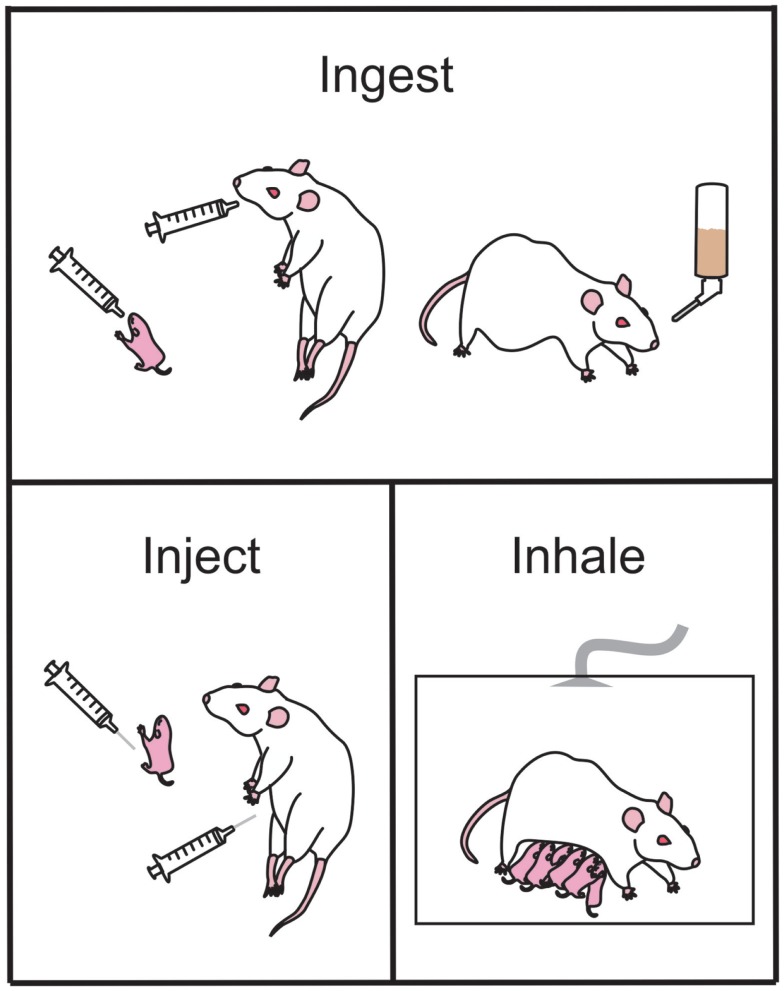
**Common ethanol administration techniques in rodents used to examine the effects of prenatal ethanol exposure in offspring**. Ethanol may be ingested by the animal via gavage administration during the early postnatal period (upper panel, left-most) or during gestation (upper panel, middle). Alternately, ethanol may be ingested as a liquid diet (upper panel, right-most). Ethanol injections (bottom left panel) can be administered pre- or postnatal for studies of exact timing of ethanol-induced damage. During the early postnatal period of offspring, the dam and litter can be placed in vapor chambers and be exposed to inhaled gaseous ethanol (bottom right panel).

The liquid diet model of ethanol exposure is one of the most commonly utilized routes of delivery in mouse and rat models and was one of the first models to be developed (83–85). Generally, food is provided to pregnant dams as a liquid diet throughout gestation in which a percentage of the calories (usually ~35%, which equates to 6.61% v/v) are derived from ethanol (Figure 1). This diet is the only source of nutrition throughout the pregnancy. Using this method, rats can consume on average 12 g ethanol/kg/day (and up to 18 g/kg/day) (25). Consumption of the diet usually begins on GD 1 of pregnancy, and the diet is introduced slowly over a three-day period (i.e., one third final ethanol concentration on GD1, two thirds of final ethanol concentration on GD2, and final ethanol concentration on GD3 and for the remainder of the pregnancy). Pair-fed control groups are often included when using this method, where an isovolumetric, isocaloric replacement (such as maltose dextrin) for the ethanol calories is used and food is restricted to that of the ethanol consumption groups (86) (see Finding the Right Control Group). The liquid diet model reliably produces BACs between 80 and 180 mg/dl in rats, i.e., a low to moderate level of exposure (15, 17, 55, 87–90), which are accompanied by neurological deficits similar to what are observed in children with FASD (see Blood Alcohol Concentration).

##### Voluntary drinking

Similar to the liquid diet model, ethanol can also be administered through the drinking water (Figure 1). This is usually achieved by training female mice or rats to voluntarily consume a saccharin-sweetened 10% ethanol solution prior to pregnancy (68, 91). Control groups receive saccharin-sweetened water only. Throughout pregnancy the rodents have *ad libitum* access to regular rat chow. Following birth, ethanol is removed from the water in a step-wise fashion to prevent ethanol withdrawal effects (68, 91). Using this paradigm, rodents tend to consume 14 g ethanol/kg/day and the BAC achieved is 120 mg/dl (68, 91).

Advantages of the liquid diet or voluntary drinking models are that the techniques are simple, less time consuming, and less labor intensive when compared to other methods. There is also much less handling of the animals associated with these procedures (a source of stress) and there is less risk of fatality. Disadvantages result because this method does not allow for the precise control over dosage or timing of ethanol exposure and this can lead to increased variability in the BAC achieved, as ethanol consumption depends on voluntary food consumption throughout the day. For example, a study by Mankes et al. (92) found that ethanol consumption of a group of 221 rats fed a liquid diet ranged anywhere between 4 and 18 g/kg/day depending on that rat (92). It is also important to remember that the liquid diet or voluntary drinking paradigms do not include alcohol exposure during the third trimester equivalent. While pregnant dams could be continued on a liquid diet during the suckling period, it is uncertain how much ethanol can cross into the breast milk and the actual dose of ethanol consumed by the pups could not be controlled for. Dams consuming ethanol during the suckling period may also be less attentive to their pups and may not engage in appropriate maternal behavior leading to social and nutritional stress [see Ref. (25) for review]. Therefore, these models are normally only used to examine exposure during the first and second trimester equivalents in the rat and mouse. Because human mothers can often be unaware they are pregnant and inadvertently drink during these periods, these models still have significant legitimacy for the human condition.

##### Intragastric intubation (gavage)

Ethanol can also be delivered directly to the stomach using an intubation method (73, 74, 78–80, 93, 94). Typically, a syringe is attached to a curved steel gavage needle, or plastic tubing, that is inserted down the esophagus to the entrance to the stomach (Figure 1). This method allows ethanol to be administered to pregnant females (ethanol is usually diluted in water or saline) and to neonatal pups (ethanol is usually diluted in a nutritional formula). An isocaloric control liquid (such as maltose dextrin or sucrose) can also be administered by gavage to control for the stress and nutritional effects of this procedure. This method is commonly used in rodents including rats (74, 78–81, 95–98) and guinea pigs (99–103), as well as primates (104–107). The dose of ethanol typically ranges between 2 and 6 g ethanol/kg/day; but produces BACs generally greater than 200 mg/dl. Often the daily dose of ethanol is divided into two administrations, given 4–8 h apart, creating two lower peak BACs in a 24 h period (42). A major advantage to this method of administration is the precise control over the dose administered and hence the peak BAC reached. A further advantage is that neonatal pups can be exposed to ethanol, allowing study of the effects of ethanol during the third trimester “brain growth spurt.” However, care must be taken to ensure that neonates adequately gain weight during the period of alcohol consumption and often a milk supplement needs to be provided to maintain healthy body weight [see Ref. (25) for review]. A significant disadvantage of intragastric intubation is that it is invasive and a very time-consuming procedure to undertake. Increased stress and higher mortality rates are also associated with this model, and individuals performing this procedure need to undergo specific training to become competent in the procedure.

##### Artificial rearing (pup in a cup)

In order to provide neonate rodents pups ethanol during the third trimester equivalent, pups can be reared artificially though a method colloquially known as “pup-in-the-cup” [see Ref. (25) for a review]. In this procedure, the pup receives intragastric ethanol, or a control solution while being maintained in a warm cup filled with nesting material in an effort to mimic the cage environment and maternal interaction early in life (108, 109). Although this method can be used to reliably administer known amounts of food and ethanol, it is invasive, expensive, and isolates each pup, removing many of the social factors that are present during normal neonatal development (i.e., presence of littermates, maternal grooming, etc.).

#### Injection

Ethanol is often administered to rodents via a subcutaneous (s.c.) (40, 110, 111) or intraperitoneal (i.p.) injection (112–114) either acutely or across multiple days during gestation (Figure 1). This method of administration is particularly useful for examining the acute effects of ethanol on distinct periods of development, and allows for a rapid increase in BAC with limited handling-induced stress. However, this method of administration does not resemble ethanol consumption in human beings and may not accurately replicate several important aspects of human PNEE. For example, i.p. injections of ethanol during the first trimester equivalent in mice result in a higher incidence of malformation when compared to the same ethanol dose delivered via intubation (114). Ethanol administered i.p. to pregnant guinea pigs was also shown to cross from the intraperitoneal space into the uterus and chorioamniotic membranes and amniotic fluid as well as being absorbed into the mothers circulation (115). This indicates that the fetus is exposed to high levels of ethanol very soon after injection, which does not accurately mimic what occurs following oral ingestion.

#### Inhalation

The inhalation mode of administration is not as commonly used as some of the other methods but a brief overview of the procedures is warranted for this review. Using this method, pregnant dams, neonatal pups, or the dam and her litter are placed in an inhalation chamber filled with ethanol vapor for several hours (116–119) (Figure 1). This method causes a rapid, reliable increase in BAC without the stress of intubation. It is also much less labor intensive than other methods and multiple animals can be in the chamber at one time. However, this method of administration does not mimic the route of intake in human beings and therefore may not be an accurate model of FASD. Additionally, the irritation to the upper respiratory tract by vaporized ethanol can be a significant factor to consider. If this method is used to expose rat or mouse pups to ethanol during the third trimester equivalent, then pups may have to be removed from their mothers for extended periods of time that may result in reduced food intake and stress associated with the separation (117), which can have lifelong effects on pups (120, 121). Finally, this method does not currently have an effective control group to account for the loss of nutrition and separation stress in the newborn pups.

#### Choosing an administration model

When deciding on the appropriate route of administration, the first issue that should be considered is the BAC we want to achieve. The easiest way to get high binge-like BACs is to inject ethanol. Using this method, stable high BACs are achieved in 45 min to 1 h following injection (111). Oral intubation with ethanol or an ethanol/milk mix can also produce high BACs with maximal effects 2 h post-injection (79, 80). The benefit of the oral intubation route of administration is that it is resembles the human condition – the ethanol is being consumed orally, and therefore enters the circulation through the same mechanisms through which it occurs when a human beings consumes alcohol. If moderate steady BACs are more relevant to the research question, then choosing a liquid diet or voluntary drinking model is more appropriate, as BACs between 80 and 180 mg/dl are usually achieved (55, 88–90, 122–128). However, there is more variability associated with this model, because an animal’s eating patterns may differ throughout the day and through each day of the pregnancy.

Another issue which needs to be considered when using many of the well established models of FASD is that ethanol is often given chronically (i.e., via a liquid diet or oral intubation) throughout gestation. This method of administration may not directly resemble the human condition. Pregnant human females are more likely to binge drink early in the first trimester, prior to discovering they are pregnant, or drink moderately on a couple of occasions each month throughout pregnancy (129). While the period of liquid diet exposure or oral intubation can be restricted, this often introduces large amounts of variability into the groups, which can make it difficult to infer the direct effects of ethanol. For example, in a recent study both the liquid diet and gavage models were utilized to expose rats to ethanol during the first (liquid diet), second (liquid diet), or third (gavage) trimester equivalent. When synaptic plasticity in the hippocampus was examined in adult animals, the variability between models was significant enough to mask differences caused by ethanol alone between the treatment groups (130).

### Finding the right control group

As well as affecting the brain, alcohol can also irritate the gut and can affect nutrient intake and absorption (131). In fact, it can often be difficult to separate the nutritional effects that accompany alcohol consumption from the teratogenic effects of alcohol alone (131, 132), and some studies suggest that nutritional deficits exacerbate the effects of alcohol (133–135) or that supplementation during the period of alcohol exposure may limit damage (131, 136). Because of the large interplay between alcohol and nutrition, having appropriate nutrition controls that help to distinguish between the deficits due to diet and the deficits purely due to the teratogenicity of alcohol are important to consider when choosing a model. A “pair-fed” control is often utilized for this purpose in most rodent models of FASD. A pair-fed group acts as a calorie-matched control group, with each animal receiving the same amount of food in g/kg/day as its matched ethanol consuming pregnant dam. Normally, a carbohydrate substance (such as maltose dextrin or sucrose) is used to account for the ethanol-derived calories in the diet. Using a pair-fed group can also control for the stress of any procedures that the ethanol group may be subjected to. For example, if an oral intubation method of ethanol administration is used, pair-fed animals can be orally intubated with an isocaloric amount of maltose dextrin/sucrose, and are therefore subjected to the same procedures as the ethanol animals.

While the use of a pair-fed control group is desirable, it should also be noted that they are not a perfect control group. They are required because animals receiving ethanol generally consume less food (and therefore less calories) than control animals (79, 90, 124, 126). However, imposing caloric restriction on naïve animals can also be perceived as introducing a stressor which can be a confounding factor in many studies (69, 124, 137). While ethanol-exposed animals eat less food voluntarily, pair-fed animals are forced to eat less and spend many hours of the day hungry. Another potential problem with this model is that ethanol has inflammatory effects in the stomach [see Ref. (138) for review]. This means that any food that is ingested may not be metabolized as efficiently, and nutrients from the food that is consumed may not be absorbed (139, 140). Unfortunately, this side-effect of ethanol consumption cannot be replicated in pair-fed animals, and therefore it is not possible to be entirely certain that the results observed are not due to a lack of absorption of nutrients. However, in mothers consuming ethanol during pregnancy, this mal-absorption would also occur, therefore the effects we see are reflective of what occurs in alcohol consuming mothers.

Rodent models using the liquid diet model of ethanol exposure, where pair-fed animals consume a liquid diet with maltose dextrin substituted for the ethanol-derived calories, show varying results in pair-fed animals, with some studies showing deficits (128, 141, 142), and others showing no differences between pair-fed animals and controls (88, 143, 144).

### Specific animal models of FASD

#### Simple systems

There are several invertebrate species that have been employed for alcohol studies. For FASD research, the microscopic nematode worm *C. elegans* is the most commonly used. While mammals offer significant advantages over invertebrates when examining brain structures or complex behaviors, simple invertebrates such as *C. elegans* can be extremely useful when examining basic biological development at the cellular, molecular, and genetic levels (145). The complete genome of *C. elegans* has been sequenced, and the simple nervous system contains only 302 neurons with 5000 synapses. Furthermore, the stages and timing of embryonic development are well characterized and a transparent egg allows for direct visualization of each of the developmental stages. A significant disadvantage to using this model is that the egg develops outside of the body and therefore alcohol exposure cannot occur as it does in human beings (via the placental membrane following oral ingestion). Instead, *C. elegans* eggs or newly hatched larvae are exposed to ethanol through bath application (145). Another disadvantage with this model is that BACs cannot be directly measured. However, if ethanol is applied at a 0.4 M concentration, previous studies in adult *C. elegans* have shown that an internal ethanol concentration equivalent to 100 mg/dl can be reached (146). In studies that have used this model to examine the effects of ethanol on development, ethanol exposure produced, in a dose-dependent manner, significant growth retardation, slowed the developmental process, impaired reproduction, and lead to early demise in the offspring (145, 147) indicating that ethanol can have similar effects on development in *C. elegans* as in human beings. Future work using this model may be able to shed light into some of the genetic mechanisms of PNEE, and whether particular genes may confer sensitivity or resistance to the toxic effects of ethanol during development (145, 147).

Simple vertebrates such as the zebrafish (*Danio rerio)* and the clawed frog (*Xenopus laevis*) are also commonly used in scientific research. These animals are cheap, small, easy to keep, have a very short developmental period, and can produce large amounts of offspring (148). Like *C. elegans*, early stage embryos have a transparent egg, and the mature zebrafish or immature *Xenopus* tadpole are also relatively transparent, allowing internal structures to be imaged very easily. Because the stages of development are thoroughly understood and can be visualized easily, it is possible to expose embryos to ethanol during very distinct and short periods of development, which can be very important for determining critical periods of ethanol exposure (149).

Also important for FASD research is the fact that the genomes of these simple vertebrates are completely sequenced and many of the genes have a mammalian counterpart. However, like with *C. elegans*, the developmental process and the physiology between these species and a human are very different. An advantage of using simple vertebrates over invertebrates such as *C. elegans* is that simple behaviors can be tested in both zebrafish and clawed frogs. This means that these animals can be used to assess functional deficits following PNEE as well as anatomical or physiological deficits (148). However, ethanol application using these organisms must still occur through bath application, with ethanol having to infiltrate the chorion of the egg, so actual concentrations of ethanol that the embryo is exposed to can be highly variable and large doses of ethanol are needed to ensure that adequate amounts cross into the embryo (binge-like exposure) (150, 151).

Studies utilizing the zebrafish or clawed frog as models for PNEE have shown that ethanol exposure during development can cause growth retardation including reduced body length, microcephaly, skeletal deficits, and eye malformation (48, 149–154) as well as cognitive dysfunction in simple behavioral tasks such as visual acuity tests (149), associative learning (54), and social behavior (155), which were apparent even in the absence of physical malformations (54, 155). These deficits were also accompanied by changes in gene expression (151, 153, 154). These effects were dependent on the dose of ethanol used and the developmental timing and length (chronic vs. acute) of exposure, with the blastula, gastrulation, and somitogenesis periods being particularly sensitive to the effects of ethanol (48, 150).

#### Rodents

Rodents are the most commonly employed models for FASD research. Rodent models are ideal for exploring basic science questions that relate to molecular biology, synaptic plasticity, and cognition. There is also a vast body of literature on rodent physiology, behavior, anatomy, development, reproduction, and teratology (25, 57). The advantages and disadvantages of each of the models as well as the main routes of exposure used are discussed in detail below.

##### Mice

Mice are the most commonly used mammals in scientific research due to the ease of care, the availability of transgenic and disease models, their short life span and their similarities to human beings in terms of genetics and basic physiology. Mouse models of FASD first began to appear in the early 1980s and seminal work by Dr. Kathleen Sulik paved the way for small mammalian models of FASD (47). The route of administration varies from study to study, with the most common models using i.p. injection (47, 113, 156, 157), s.c. injection (111, 158, 159), voluntary drinking paradigms (91, 160, 161), liquid diets (162–164), or oral intubation (165). Most studies employ chronic exposure paradigms (i.e., throughout pregnancy or throughout the third trimester equivalent), but intermittent exposure is also common, particularly in studies where the i.p. route of ethanol administration is used, and where critical periods of vulnerability are being examined (47, 113, 158, 159, 162, 166, 167). The BACs achieved in most studies range between 80–180 mg/dl (for voluntary drinking or liquid diet) and over 200 mg/dl for studies where i.p. injections or oral intubation is used. C57BL/6 is the most common strain of mouse used, but other similar strains are also employed. The ability to genetically manipulate mice can be a huge advantage and many studies into the genetic components associated with FASD have utilized mice as a model (160, 165, 167, 168). A disadvantage with using mice is that the third trimester equivalent of development occurs following birth (see Developmental Timing of Ethanol Exposure). To overcome this, many studies will administer ethanol during the early postnatal period (third trimester equivalent, PND 1–10, see Artificial Rearing), however, issues arise with this method because ethanol exposure occurs outside of the confines of the placental barrier and kinetics and metabolism may be fundamentally different when compared to what happens *in utero*. Despite this, mice are still commonly used, and many common features of FASD that are observed in human subjects are also observed in mice, including craniofacial abnormalities (47, 113, 157), eye malformation (47), growth retardation (162, 163, 166), and cognitive deficits (111, 156, 159–161, 163, 165) [see Ref. (169) for review]. These deficits have been observed across the lifespan (i.e., in neonatal, adolescent, adult, and aged animals) and with all routes of exposure, although the severe growth malformations and facial deficits are often not apparent in models with lower BACs. As well as fundamental studies on the underlying pathologies associated with PNEE, mouse models are also useful for examining potential therapeutics (156).

##### Rats

Like mice, rats are commonly used as models of FASD. One of the more obvious advantages of rats is their larger size, which makes handing and sampling procedures easier. Rat models also offer an advantage over mouse models because more sophisticated behaviors, including tests of learning and memory and executive function (see Behavioral Manipulations) can be examined more easily in rats, whereas mice have a more limited behavioral repertoire. Like mice, rats have a short lifespan, a gestational period that is analogous to the first two trimesters of human gestation, and neither species requires very sophisticated housing facilities normally (see Developmental Timing of Ethanol Exposure).

Many routes of ethanol administration are used in rat models of FASD: chronic exposure (i.e., throughout gestation) producing moderate stable BACs occurs with liquid diet and voluntary drinking paradigms (55, 69, 87–90, 122–128, 170–180), or if high BACs are preferred oral intubation can be used, either during the gestation period (181), the third trimester equivalent only (56, 95–98, 182–187), or through all three trimester equivalents (78–80). Vapor inhalation (188, 189) is seldom used in current protocols and injection of ethanol i.p. or s.c. does not occur as commonly in rat models and tends to be reserved for mouse models where the effects of ethanol on neuroanatomical features are examined (47, 67, 157).

Like with mice, all the hallmark features of FASD have been demonstrated in rats including growth retardation (174, 188), structural abnormalities (31, 79, 80, 181, 183, 190–193), CNS dysfunction (88, 89, 124, 179, 180, 189, 194), and cognitive deficits (55, 56, 78, 95, 174–178, 184–187). Many of the impairments observed are dose and timing dependent, but are observed across the lifespan and with all routes of ethanol administration. It is also possible to screen potential therapeutics in rat models of FASD and many treatments given either concomitantly with ethanol or following ethanol exposure (i.e., by supplementing offspring after birth) show promise for the mitigation or reversal of some of the cognitive impairments associated with FASD (56, 89, 90, 122).

##### Guinea pigs

Guinea pig models are utilized in some laboratories as they offer the advantage of being a true *in utero* exposure model because the three trimester equivalents of brain development largely occur during gestation (as opposed to the rat/mouse where the third trimester equivalent is during the early postnatal period). The oral intubation administration route is commonly utilized in guinea pig studies with the dose of ethanol ranging from 3 to 6 g/kg/day (99, 102, 103, 195, 196). In some studies, ethanol administration begins prior to gestation (196) but in the majority of studies ethanol administration begins on GD 1–2 (100, 102, 103, 197, 198). In most studies [excluding (196)], a nutrition/stress control group (which receive sucrose by oral intubation) was included. Results from these studies have indicated that PNEE can cause structural (101, 196–198), functional (103), and cognitive deficits (102, 103, 195) that mimic the human condition. These deficits were observed in neonate (101, 102, 196, 198), adolescent (197, 198), and adult (102, 103, 197, 198) animals. There has been only one study where ethanol administration has been restricted to the third trimester equivalent (classified as GD 43–62) (99) and surprisingly, hippocampal synaptic plasticity and spatial learning were not significantly affected in adult animals even with BACs of 245 mg/dl (99). Recently, studies utilizing the guinea pig model have been exploring the idea of biomarkers for FASD. Specifically, the accumulation of fatty acid ethyl esters, which form during non-oxidative metabolism of ethanol, in the hair may be a useful indicator of PNEE (199). The advantage of using the guinea pig model for this research is that guinea pigs are the only rodent species that are born with neonatal hair. This line of research may result in the guinea pig model being more widely used in the FASD field. A drawback in using guinea pigs is that the litter size is much smaller than in rats/mice and the longer gestation period can increase the time and costs of a project. Furthermore, guinea pigs may be more difficult to use for behavioral testing as they are not naturally exploratory and may not perform as well as rats in many behavioral tasks (200).

#### Primates

Because primates are our closest evolutionary ancestors, primate models of FASD are considered a “gold standard.” Developmental gestation and length resembles human pregnancy, and more importantly, primates can be used to study more sophisticated behaviors than are possible in rodents or other animal models (201). However, primate research is time consuming (pregnancy length is similar to human beings), expensive, and ethical approval can be difficult to obtain. Because of this, there are very few studies of PNEE that have been conducted in primates and those studies that have been done usually have a very small sample size and there are wide variations in ethanol dosage and administration. For example, one of the first studies conducted by Elton and Wilson (202) allowed four pig-tailed macaques (*Macaca nemestrina*) to consume an orange-flavored ethanol solution prior to conception and throughout pregnancy. While three of the monkeys drank very little of the ethanol and had apparently normal infants, one of the monkeys consumed large amounts of the ethanol throughout her pregnancy and her infant was noted to be hyperactive and tremulous (202). The majority of primate studies utilize the oral intubation method for administering alcohol (104–107), with many studies only giving alcohol once weekly rather than daily, which may more closely resemble human drinking patterns during pregnancy (104, 106, 107). Dosage of ethanol ranges from 0.3 to 5 g/kg and while BACs are not often reported in these studies, in those where they are reported they range from 150 to 250 mg/dl (104, 203). Voluntary drinking paradigms are also used in some studies (0.6 g ethanol/kg/day), and much lower BACs are achieved (20–50 mg/dl) (204–206). There is a large variation in the period of ethanol exposure; in some studies ethanol is administered throughout pregnancy (202, 204, 207), in some it starts after the first month of pregnancy (106) and in others it is intermittent (104, 105, 205). Results from primate studies have shown that ethanol exposure during development produces growth retardation (104–106) as well as behavioral deficits in adolescence and adulthood (104, 105, 204–206) similar to those observed in human beings with FASD.

### Summary

There are many different factors to consider when choosing a model to conduct research on FASD. The animal model that is chosen should reflect the specific research question that is to be answered. Depending on what is to be examined, each model offers its own advantages and disadvantages. Peak BAC, developmental timing, route of administration, and stress and nutrition controls should also be considered. Simple invertebrates and vertebrates such as *C. elegans, Xenopus*, and zebrafish can be excellent tools for examining the effects of ethanol at a genetic level or on very specific stages of development. Rodents are more commonly used for translational research where the effects of therapeutics can be examined for future use in a clinical population. Non-human primate models are gold standard when it comes to examining complex behavior, but studies are often limited due to small sample sizes, large costs, and time constraints.

## Behavioral Manipulations

There are many documented behavioral manipulations that have been used to characterize the functional consequences of PNEE in animal models that often correlate with known human dysfunctions [see Ref. (208) for a review of human behavioral work]. With respect to animal models, behavioral experiments are necessary tools when assessing the use of novel therapeutic approaches for PNEE offspring. Here, we will outline five major classes of behavior, including several key behavioral tasks where performance is affected by prenatal ethanol ingestion, injection and inhalation.

### Motor skills

The cerebellum is a region of the fetal brain that is particularly vulnerable to damage by ethanol *in utero*. Motor hyperactivity is often reported in children with FASD. Children with FASD perform poorly on fine motor coordination and reaching tasks (209) and have deficits in postural balance (210). Recently, children diagnosed with FASD were found to have poor saccade accuracy (211), a task dependent on the cerebellum (212). Motor performance can readily be evaluated in animal models using standardized tasks that include the rotarod, runway, directed reaching, and gait analyses.

In rat pups exposed to ethanol via intubation throughout all stages of pregnancy and during the early postnatal period, the overall volume of the cerebellum and Purkinje cell (PC) numbers were reduced (213). Others have identified the third trimester equivalent as a period of particular vulnerability for PCs (214). PC density is reduced in PND 10 rat offspring exposed to ethanol (gestational intubation) and the ultrastructure of this neural population is modified, indicating a delay in cellular development (215). The widely reported damage to the cerebellum has observable, functional consequences on motor-related behaviors.

**Figure 2:**
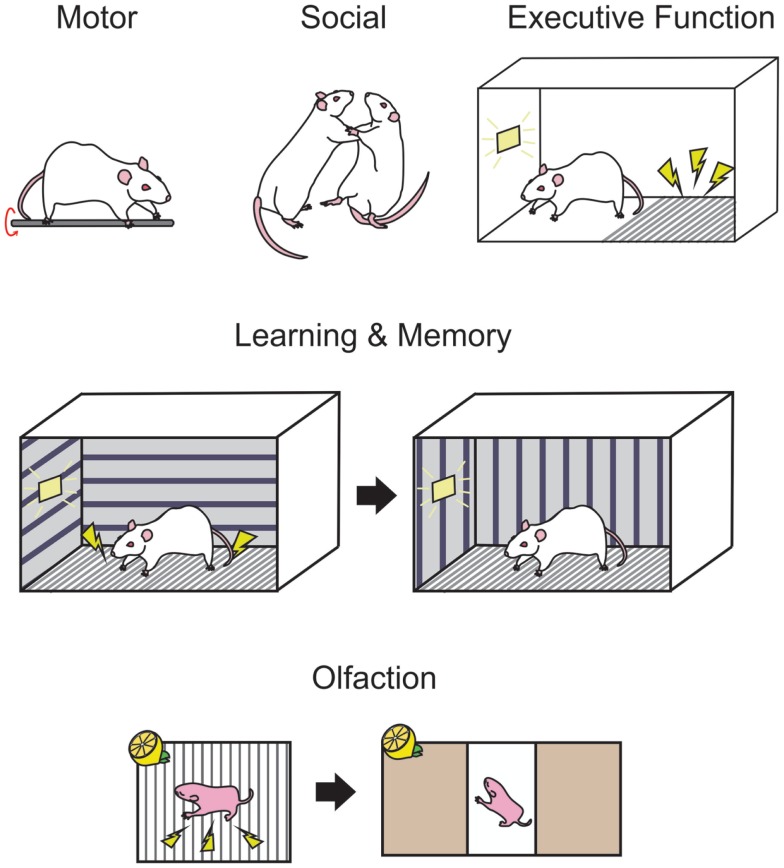
**Standardized behavioral measures are used in rodents to examine the functional consequences of developmental ethanol exposure**. Motor performance in rodents may be measured on tasks such as the rotarod (upper panel, left-most) where the animal must balance and run on a rotating rod. Social interactions, such as evidence of aggressive behavior can be deduced by observations of wrestling, rearing, and pinning (upper panel, middle) when two conspecifics are paired. Passive avoidance (upper panel, right-most) is a measure of executive behavior in rodents where the animal must learn to inhibit exploratory behavior in order to avoid a shock for the duration of a trial, as indicated by a light. Trace fear conditioning (middle panel) is a hippocampal-dependent behavior where the animal is trained in a context where a signal such as a light indicates a footshock, then after a delay in placed in a novel context and freezing responses can be measured while the light is presented without the footshock. Associative olfactory memories (bottom panel) are formed when an odor, such as lemon is paired with a stimulus such as a footshock and may be tested in a two choice preference chamber, where orientation toward or away from an odor can indicate the presence of a memory for that odor.

Behavioral tasks such as the rotarod, runway tasks, and gait analysis software may be used to examine damage to the cerebellum and related motor structures. In the rotarod task, a rodent is placed in a rotating bar and is required to run on the rod for as long as possible. The rotational speed of the bar can be increased, and the experimenter can then measure the duration of time that the animal can remain on the bar at various speeds (see Figure 2). In rodents, it is thought that the motor deficits caused by PNEE are most apparent early in life, and in most cases unseen at adulthood. Bond and DiGiusto (gestational liquid diet) showed these age effects with the anticipated motor hyperactivity in PND 28 and 56 rat offspring, while seeing no evidence of motor impairment at PND 112 (216). Similarly, adult rat offspring (intubation GD 7–20) shows no evidence of motor dysfunction or hyperactivity on a rotarod or open field task (217). During this early window of observation, rats (gestational liquid diet) have been found to be ataxic, exhibiting asymmetrical gait, shorter stride length, and greater step angle than their respective controls (218). Young mice (<PND 60, gestational ethanol in drinking water) perform poorly on the runway and rotarod tasks (219). However, when ethanol administration was restricted to the postnatal period (intubation PND 4–9) as adults (>PND 70), these animals perform poorly on an eyeblink conditioning task, a form of classical conditioning where a light is paired with a puff of air on the eye, causing the animal to blink (220). The impaired performance on this hippocampal-independent task is thought to be due to ethanol-induced damage to the interpositus nucleus of the cerebellum (221). Thomas and colleagues (182) examined the specific timing of postnatal exposure to ethanol (via gastronomy) in relation to cerebellar damage and motor performance. This study identified PND 4/5 as a critical period for ethanol exposure where the greatest deficits could be seen on a parallel bar task at PND 30 and 52, where the width between bars that the animal was required to cross over was gradually widened. This time point also produced the greatest decreases in cerebellar and brainstem weights at PND 55 (182). Others have shown that when ethanol is restricted to the postnatal period (intragastric ethanol PND 2–10), motor hyperactivity in rats persists into adulthood, at least until PND 91 (74).

These studies stress the importance of considering the timing of ethanol administration, the age of the offspring when conducting motor behavior studies, and highlight the need for additional studies in this area in aged animals.

### Executive function

Executive functioning is the ability to use appropriate problem solving in goal-directed behaviors, and includes behaviors such as response inhibition, working memory, and set shifting. These functions have long been thought to be dependent on frontal lobe structures [see Ref. (222) for review] such as the prefrontal cortex, though some argue that extra-frontal-lobe structures may also be involved [see Ref. (223) for review]. In human beings, these behaviors can easily be measured through standardized tests, and they appear to be gravely impacted by prenatal alcohol use [see Ref. (224) for a review]. Children with FASD have difficulties inhibiting responses on the Stroop test (225), a task where an individual must inhibit the natural tendency to read words, being required instead to state the color of the font. In addition, these individuals have difficulty in suppressing saccade responses in visual tasks while waiting for the proper initiating signal (211) and exhibit poor working memory when asked to recall digit spans backwards (226). On the Wisconsin card sorting task, where the subject must detect, use, and change card sorting strategies, individuals with FASD make more errors related to shifting sort strategies (227). In rodents, executive function tasks are complex and a single task often requires the use of response inhibition, working memory, and set shifting among others.

Response inhibition tasks require the subjects to inhibit responses that the organism may be naturally predisposed to perform in particular environments. For example, in rodents, passive avoidance is a task commonly used to show response inhibition. In these tasks, the rodent is placed in a box on a “safe” area, adjacent to a grid floor that will provide a shock if the animal steps onto the grid within a trial. The animal must learn to inhibit the natural tendency to explore new environments and remain on the “safe” side of the test chamber for the entire trial (see Figure 2 for a schematic). PNEE rats prenatally exposed to ethanol (liquid diet GD 6–16) show impairments in these passive avoidance tasks, at both PND 18 and PND 41–53 (228). In a large rat study, offspring exposed to ethanol (liquid diet GD 5–20) again exhibited impaired passive avoidance of a shock at both PND 17 and PND 48, but not at PND 114, and took longer to spontaneously alter their exploratory strategy on a T-maze where the animal would be confined after visiting a particular arm when tested at PND 16 and PND 63 but not at PND 112 (229). Rats exposed prenatally to ethanol (liquid diet, GD 1–20) have fewer cells in layers II and V of the medial prefrontal cortex, which was correlated with poor performance on a reversal learning task in adulthood [>PND 90; Ref. (230)].

Working memory is a short form of memory where information from a recent experience must be used to perform the appropriate response on a following trial or task. Working memory is a form of memory that is known to primarily require the functional activity of the prefrontal cortex [see Ref. (231) for review of human beings working memory and see Ref. (232) for a review of the cellular mechanisms of working memory], differentiating it from other forms of memory discussed in Section “Learning and Memory.” Behavioral tasks that evaluate working memory include delayed matching to sample tasks where a stimulus is provided, followed by a delay then a choice between multiple different stimuli. In these experiments, the organism must remember the initial stimulus then pick the matching stimulus when given a choice after the delay, and is readily adapted for rodent, non-human primates, and human beings. For rodents, the task can be modified to a delayed matching-to-place task in a Morris water maze (MWM), where a platform is located in an arm during a search trial, then after a delay the animal must return to the location of the platform during the search session. When ethanol administration occurs in the third trimester equivalent (gastronomy PND 6–9), these rats perform poorly on the matching-to-place task, at PND 35, PND 105, and PND 180, when the delay between the search and test trials is 2 h (233) though this task requires both intact working and spatial memory.

Set shifting is a complex task that can readily be performed by human beings and non-human primates, with variable evidence from rodents. In rats, set shifting tasks are not as well established as human beings and non-human primate work. In delayed non-matching-to-sample tasks, the subject not only requires functional working memory and inhibitory control but also set shifting where the organism must be able to observe the sample stimulus then shift their attention to choose the non-matching option during the subsequent test trial. In one rat study of delayed non-matching-to-sample, adult PNEE animals (liquid diet GD 1–22) showed no impairments in set shifting (234). Future rodent work in this area may use a unique behavioral task adapted from primate studies (235) in order to fully understand how set shifting may be altered by PNEE in rodents and shed light on the underlying neural substrates for these behaviors. In a study of rhesus monkeys exposed to ethanol (GD 5 – parturition, voluntary drinking), the 32–34 month old offspring had difficulty acquiring a delayed non-matching to sample task (204).

When using animal models to examine the effect of PNEE on executive functions, it is critical to design appropriate tasks for the model in question. Tasks used for one particular species may not be easily applied to other without modifications for the species in question.

### Learning and memory

The damaging effects of PNEE on learning and memory have been reliably reported in many species. Here, we will focus on hippocampal-specific learning and memory behaviors in rodents and in human beings. In spatial object memory tasks where a child must remember the location of multiple objects on a semi-random grid, children with FAS were unable to recall objects after a delay and exhibited distorted spatial array when asked to recall where the objects were (236). Additional work with human beings with FASD is necessary to understand the manifestations of neural damage caused by PNEE. Future studies of spatial memory may utilize virtual 3D object-recognition tasks where the subject can undergo PET or fMRI scans while virtually exploring a space (237) as in (238).

In rodents, hippocampal-dependent memory can be assessed in a variety of behavioral tasks including tasks such as the MWM and fear conditioning. PNEE-induced hippocampal damage has been widely reported in rodents (193, 194, 239), for review see Ref. (25, 86).

The MWM is a standard task where a T, plus or open field maze can be submerged in cloudy water. A platform can then be hidden below the surface, and visual detection of the rodent when swimming in the maze. The animal must swim to explore the maze and find the submerged platform to escape the water in multiple training trials where variables such as latency to the platform, swim speed, and distance traveled to platform can be measured. As described above, the MWM can be adapted for many functions, such as delayed matching-to-place (233), which are readily learned by healthy rodents. However, PNEE rodents exhibit significant impairments on this task [rats: liquid diet GD 1–22 (55, 141, 175) and intubation PND 4–9 (56, 95, 184–186); guinea pigs intubation GD 2–67 (102)].

Fear conditioning is a behavioral task that is both easily implemented and readily learned by rodents. Trace fear conditioning occurs when an unconditioned stimulus (US), such as a footshock, follows a conditioned stimulus (CS) such as a tone or a light. Following multiple training sessions, the animal is tested in a novel context similar to the training context and freezing responses are recorded in response to presentation of the CS alone (see Figure 2). PNEE rats perform poorly on this task when ethanol is given in the third trimester equivalent [intubation PND 4–9 (240)], with poorest performance observed when ethanol administration occurred from PND 4–6 [intubation (241)].

Other forms of hippocampal memory are impaired by PNEE in rodents. Popovic and colleagues (177) subjected PNEE offspring exposed to gestational ethanol in a liquid diet and/or the early postnatal period to an extensive battery of memory tasks to evaluate performance in spatial learning, orientation, and simple and more complex object recognition. Generally, ethanol-exposed offspring performed poorly, though the impairments in these animals became increasingly evident as the task difficulty increased, with animals treated during the early postnatal period performing worse than others (177).

### Social behavior

Social behaviors in human beings and non-humans alike are complex interactions between genetics, early life experiences, and later social learning that can be altered by PNEE [for a review, see Ref. (242, 243)]. For human beings, appropriate behavior in a social context is critical for societal integration, therefore, it is critical to consider that PNEE can shape lifelong behavior, and that FASD is not simply a childhood disorder as highlighted by Streissguth and colleagues (16) in a longitudinal study examining childhood, adolescents, and adults (16).

Social dysfunctions in human beings with FASD are apparent early in life with altered sleep patterns, increased irritability, and feeding difficulties during infancy (244). Similarly, neonatal rats exposed to ethanol in the early postnatal period (gastronomy PND 2–12) take longer to attach to the nipple and spend less time suckling than controls (245), emit more vocalizations on PND 5 when separated from the dam after pre- and postnatal ethanol exposure [intubation GD 1–22 and PND 2–10 (246)] and are not retrieved by the dam as quickly as unexposed pups [drinking water GD 0–30 (247)]. These negative early life experiences can play a role in shaping social development long term.

For human beings, other social behavioral problems associated with fetal ethanol exposure become apparent at school age. When matched with unexposed children with low verbal IQs, children with FAS have poor coping skills and interpersonal relationship skills according to the Vineland adaptive behavior scale [VABS (248)], performing three standard deviations below the norm for their age. Others have also reported increased aggression in children with FAS (249). In juvenile PNEE rats (liquid diet GD 6–20), the sexually dimorphic play behaviors were reversed where males displayed female behaviors and vice versa (250). Prior to puberty, ethanol-exposed rats (intubation GD 6–19) exhibit more play behavior though males that are more aggressive (see Figure 2 for a schematic) following puberty than unexposed controls (251). It must be noted, however, the great differences between the complexity of social behavior between human beings and rodents at this age and beyond when drawing parallels between the two species.

Unlike other previously discussed behaviors, disruptions in the social behaviors of adult human beings and rats have been readily shown. In adolescent and adult human beings exposed to ethanol *in utero*, whose average chronological age was 17, the average adaptive functioning as measured by the VABS was equivalent those of a 7-year-old healthy child (16). In this same study, all adolescents and adults were classified in the significant and intermediate categories of the maladaptive behavior section of the VABS including behaviors such as social withdrawal and teasing or bullying of others. In a report on secondary disabilities associated with FASD, Streissguth et al. (252) reported that of adult females exposed to ethanol *in utero*, 40% had drank alcohol while they were pregnant, and over 50% of the children had been removed from the care of the mother. Difficulties in parenting have also been observed in rats that drank ethanol throughout gestation (253). In this study, females exposed to ethanol mother failed to retrieve pups removed from their nests, a task normally accomplished in a short time by control animals. The researchers also observed disorganized and distracted behavior in the mothers. For instance, dams might start carrying a pup part of the way toward the nest, but then drop it and be distracted by self-grooming, eating, or drinking and forget about the retrieval effort (253). Adult males also show disrupted social behaviors at adulthood. Male rat offspring exposed to ethanol prenatally spend less time sniffing other rats at PND 90 than those exposed to maternal saccharin water [gestational ethanol in drinking water; Ref. (254)] and display more aggressive behaviors, including attacks, tail rattling, and chasing in the presence of conspecifics (255). These findings in both rodents and human beings stress that the effects of FASD do not exist in childhood alone, and that they can have effects on the next generation of offspring.

### Olfaction

Olfaction is a complex sense that has recently become of special interest in the area of neurodevelopmental diseases in human beings as early indicators of disease onset, permitting for early intervention [see Ref. (256) for a review]. An early neuropathological report noted significant damage to the olfactory bulbs and stalks in children and fetuses prenatally exposed to alcohol (257) though few studies have examined the functional consequences of this damage. Olfactory abilities can readily be tested in many organisms, from human beings with “Sniffin” sticks (258) to *Drosophila* [see Ref. (259) for a review]. A recent study (260) used two sensory profiling measures filled out by caregivers to examine the sensory abilities of children with FASD. They found that children with FASD have under responsive smell and taste, though the two variables were combined in these forms. In the first study of its kind, children and adolescents exposed to ethanol *in utero* were administered the San Diego Odor Identification Test, where the child is presented with common household odors such as chocolate and peanut butter and must name the odor, revealing significant impairments in the identification of these odors (261). These findings in human beings are compelling, raising questions about the ability for human beings with FASD to discriminate between similar and different odors. Others have reported that fetal ethanol exposure increases infant reactivity to the smell of ethanol after birth, indicating some prenatal sensory memory that persists after birth (262, 263).

Olfaction is the primary sensory modality in rodents and has been extensively studied in the context of memory [for a review see Ref. (264)] and odor identification and discrimination [see Ref. (265) for review]. The olfactory circuitry is susceptible to damage from prenatal ethanol with consistent reports of olfactory bulb damage following various ethanol administration methods in mice [drinking water GD 0–26 (266); injection GD8 (67)] and rats [gastronomy PND 4–9 (267)]. Odor memory can be examined through classical conditioning tasks where an odor can be paired with either an appetitive or aversive stimulus followed by examining the orienting response of the animal to an odor. These tasks can easily be carried out early in life, by pairing an odor with tactile stimulation (268) or a footshock (269) among others (see Figure 2). These tasks can be modified for use in juvenile and adult rodents in odor operant boxes, or olfactometers, where the delivery of an odor signals an action for the animal, such as a nosepoke, in order to receive a water reward (270). These olfactometers can also be used to examine odor discriminative abilities in rodents. Odor memory in early life is impaired by prenatal ethanol (liquid diet, GD 6–20) where a PND 3 rat pup is unable to learn aversive (odor + footshock) and appetitive (odor + milk delivery) odor association tasks (271). Interestingly, the impairment in odor associative memory is not apparent at adulthood in an aversive odor association (271). Mice exposed to ethanol *in utero* (drinking water GD 0–26) have poor discriminative abilities when given similar odors in odor mixture studies though odor associative memory remained intact (266). As with human beings, neonate rats exposed to ethanol *in utero* (liquid diet GD 5–22) where ethanol odor presentation at P15 elicits an altered behavioral response to the odor compared to controls (272).

Disruptions in olfactory memory and odor identification and discrimination as a result of PNEE require more extensive behavioral work to understand how the olfactory circuitry is selectively damaged by ethanol *in utero*. Further behavioral studies in this area are required; though with extensive information available regarding healthy olfactory processing this is a viable area of study for the future.

### Summary

The study of the effects of PNEE on offspring has produced extensive evidence of behavioral disruption across multiple neural systems. When describing the damage caused by PNEE, one must consider the interactions between these systems at the behavioral level and therefore make careful choices when designing animal experiments. Together, human beings and animal behavioral impairments can shed light on potential neural targets of or vulnerabilities to PNEE.

## Conclusion

Fetal alcohol spectrum disorder remains a prevalent problem in our society (7), though there are a great deal of laboratories around the world delineating the mechanisms behind the teratogenic effects of ethanol and the underlying biochemical, molecular, and genetic events that lead to the cognitive deficits characteristic of FASD. Human beings work has identified diagnostic criteria for FASD, which has permitted the proper diagnosis of more individuals that require intervention. Animal models have also been invaluable for this body of work particularly because they allow us to examine different drugs and supplements for their potential therapeutic properties on both neural structures and observable behavior. It is critical for both fields to consider the potential lifelong implications of FASD, as there is a gap in what is understood of PNEE in adults and particularly in aged populations. Moving forward, translational research linking human beings and animal work is imperative in order to paint a vivid picture of damage caused by PNEE and to eventually find a way to overcome some of the devastating effects of PNEE.

## Conflict of Interest Statement

The authors declare that the research was conducted in the absence of any commercial or financial relationships that could be construed as a potential conflict of interest.

## Abbreviations

ARBD, alcohol-related brain defects; ARND, alcohol-related neurological disorder; BAC, blood alcohol content; CNS, central nervous system; EDC, ethanol-derived calories; FAS, fetal alcohol syndrome; FASD, fetal alcohol spectrum disorder; GD, gestational day; i.p., intraperitoneal; IQ, intelligence quotient; MWM, morris water maze; PC, Purkinje cell; PND, postnatal day; PNEE, prenatal ethanol exposure; s.c., subcutaneous.
